# pyGenClean: efficient tool for genetic data clean up before association testing

**DOI:** 10.1093/bioinformatics/btt261

**Published:** 2013-05-06

**Authors:** Louis-Philippe Lemieux Perreault, Sylvie Provost, Marc-André Legault, Amina Barhdadi, Marie-Pierre Dubé

**Affiliations:** ^1^Montreal Heart Institute Research Center, Beaulieu-Saucier Université de Montréal Pharmacogenomics Centre, 5000 Bélanger Street and ^2^Université de Montréal, Faculty of Medicine, 2900 chemin de la tour, Montréal, Canada

## Abstract

**Summary:** Genetic association studies making use of high-throughput genotyping arrays need to process large amounts of data in the order of millions of markers per experiment. The first step of any analysis with genotyping arrays is typically the conduct of a thorough data clean up and quality control to remove poor quality genotypes and generate metrics to inform and select individuals for downstream statistical analysis. We have developed *pyGenClean*, a bioinformatics tool to facilitate and standardize the genetic data clean up pipeline with genotyping array data. In conjunction with a source batch-queuing system, the tool minimizes data manipulation errors, accelerates the completion of the data clean up process and provides informative plots and metrics to guide decision making for statistical analysis.

**Availability and implementation:** *pyGenClean* is an open source Python 2.7 software and is freely available, along with documentation and examples, from http://www.statgen.org.

**Contact:** louis-philippe.lemieux.perreault@umontreal.ca or marie-pierre.dube@statgen.org

## 1 INTRODUCTION

Genome-wide association studies and similar designs typically rely on the use of massive amounts of genotype data covering the genome of thousands of study participants. Before proceeding to statistical analysis with genotyping array data, quality control (QC) and data clean up are usually performed to identify poorly performing samples and failed genotypes and to produce the analysis set according to set genetic ancestry criteria. This is particularly important for association tests, which can be sensitive to even small sources of systematic or random errors. When joined with large sample size and a large number of genotyped markers, small errors can cause loss of statistical power and spurious associations. Hence, a thorough QC is required, even though it might become computationally intensive (Turner *et al.*, 2011). PLINK (Purcell *et al.*, 2007) is a tool widely used for quality assessment (Anderson *et al.*, 2010; Laurie *et al.*, 2010; Turner *et al.*, 2011), providing multiple quality metrics about markers and samples that allows efficient data management. Even though PLINK is a complete toolset, it lacks automation, and some steps require a considerable amount of manual tuning. *pyGenClean* automates the QC procedure using PLINK, while providing the user with multiple summarization files and visual aids for quick identification of quality issues. It also allows for the parallelization of steps for servers with a DRMAA-compliant distributed resource management system. The tool consists of multiple stand-alone scripts that are linked together via a main script and a configuration file, the latter facilitating user customization.

## 2 METHODS

**Fig. 1.**
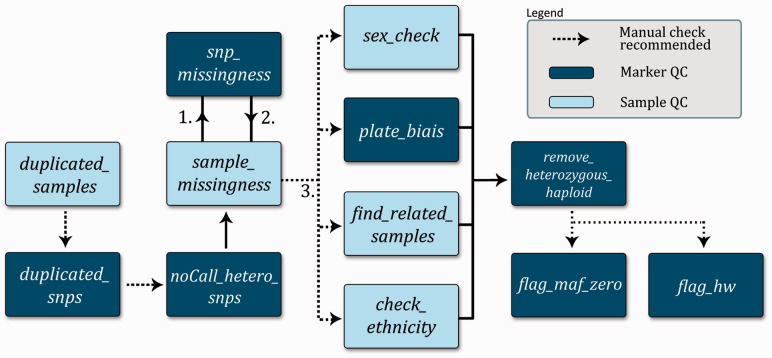
Proposed data clean up pipeline. Each box represents a customizable stand-alone script with a quick description of its function. Optional manual checks for go-no-go decisions are indicated. Numbers represent the ordering of the cyclic part of the pipeline

The QC pipeline was designed to respond to the needs of users for quality assessment tools of both the samples and their markers, historical process logs, speed and parametrization. Different QC protocols have also been previously described in the literature (Anderson *et al.*, 2010; Laurie *et al.*, 2010; Turner *et al.*, 2011). Our QC pipeline is constructed from a set of script modules, which can be parameterized and ordered according to specific project needs. Figure 1 displays the recommended pipeline structure, designed to optimize the QC and minimize sample loss. The procedures can be separated in two classes: marker and sample QC procedures.

The **marker QC** consists of seven scripts [Fig. 1 (in dark blue, see online version)]. The duplicated_snps module finds duplicated markers according to their chromosomal location (even if they have unique IDs). It computes the completion and the overall concordance of each duplicates and evaluates the possibility of merging the data of the duplicate markers when concordance criteria are met while zeroing out discordant genotypes. As multi-allelic markers would appear to be duplicated in the dataset, the script will preserve incompatible duplicates. The noCall_hetero_snps module removes any marker with a heterozygosity rate (excluding the MT markers) or a missing rate of 100%. The snp_missingness module removes all the markers with a missing rate that is higher than the user-defined threshold. The plate_bias module removes marker showing significant plate bias as assessed by the comparison of allele frequencies from one plate to all others. The remove_heterozygous_haploid module zeroes out heterozygous haploid markers. The flag_maf_zero module flags markers with a minor allele frequency of zero. The flag_hw module flags markers that fail Hardy–Weinberg equilibrium.

**Fig. 2.**
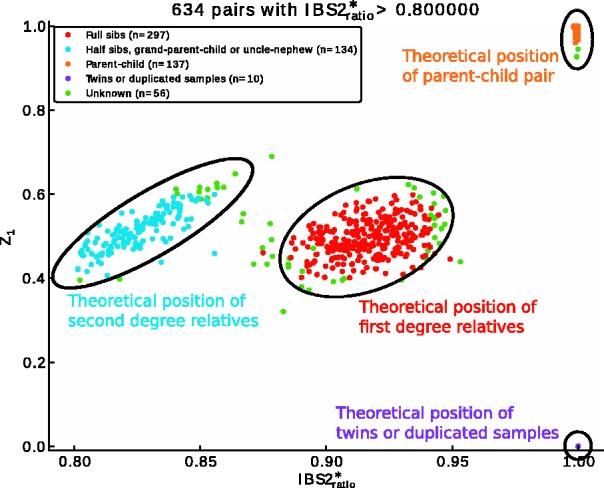
*Z*_0_ in function of 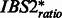 showing sample relatedness

The **sample QC** steps consist of five scripts [Fig. 1 (in light blue, see online version)]. The duplicated_samples module helps in comparing duplicated samples with identical identifiers (IDs). As the PLINK program requires unique IDs, input files for this script consist of transposed PLINK pedfiles (separated by tabulations), and the script performs the required computations. It computes the completion and the overall concordance of each duplicates and evaluates the possibility of merging the data of the duplicate IDs when concordance criteria are met and by zeroing out discordant genotypes. The sample_missingness module will remove all the samples that have a missing rate higher than a user-defined threshold. The sex_check module uses PLINK’s check sex options to compare gender registered in the input files and the gender deduced by their X chromosome heterozygosity rate. Using all samples, the script also produces a graph showing the summarized Y intensities in function of the summarized X intensities of all samples, highlighting problematic samples (Laurie *et al.*, 2010; Turner *et al.*, 2011). The script also produces a plot showing the overall log R ratio and B allele frequency (BAF) for both X and Y chromosomes of problematic samples, helping in the identification of allelic imbalance (Laurie *et al.*, 2010) and in estimating the number of X and Y chromosomes. Finally, the script computes the heterozygosity rate on the X chromosome and the number of missing calls on the Y chromosome, helping in the resolution of possible gender mix ups. The find_related_samples module uses PLINK’s genome option to estimate the relatedness of study participants using identity-by-descent and identity-by-state for each sample pairs. It finds the possible degree of relatedness using the Cotterman coefficients as estimated by PLINK (*Z*_0_, *Z*_1_ and *Z*_2_). The script also computes the 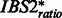 to produce two plots of *Z*_0_ and *Z*_1_ in function of 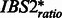 (above a user defined threshold) (Stevens *et al.*, 2011). Those plots provide visual support for the identification of sample pair relatedness (Fig. 2). Finally, the script offers the possibility of randomly selecting one sample of each related group, excluding other related samples from the final dataset. The check_ethnicity module uses the PLINK program to compute the multidimensional scaling (MDS) values of the samples. This method requires a pairwise identity-by-descent matrix to be computed, which can be computationally demanding. Using parallelization, the *pyGenClean* script efficiently computes the MDS values using PLINK and, with the addition of reference populations (such as CEU, YRI and JPT-CHB), will find outliers with respect to a user-defined reference population and will create MDS graphs. The outlier’s detection script uses the standard deviation of each cluster found by a K-Means algorithm.

Two additional scripts are provided for the automation of the QC pipeline. The first one, subset, helps to subset the dataset by excluding or selecting a set of markers or samples. The second one, compare_gold_standard, compares the current dataset with a gold standard. For example, if some samples from the 1000 Genomes Project were genotyped, the script compares the study genotypes with those of the reference 1000 Genomes Project data. If needed, markers are flipped according to their minor allele for comparability.

## 3 APPLICATION

A dataset comprising 6528 samples (including multiple duplicates of four HapMap samples and internal control samples) genotyped at the Beaulieu-Saucier Pharmacogenomics Centre on the Illumina HumanOmni2.5Exome BeadChip (2 567 845 markers including 42 822 duplicated markers) was processed with *pyGenClean*. A parameter file, as described in Figure 1, was created and used on a cluster with 10 nodes of 8 Intel® Xeon® CPUs at 2.40 GHz (with hyper-threading) and 47 GB of random access memory for each node. The sample_missingness module was run two times (with 10 and 2% of missing calls thresholds), and the snp_missingness module was run in between to minimize data loss. To optimize computation speed, the duplicated_samples module was run independently of the others, and some script was run in parallel on the cluster. All other scripts were run with default parameters.

After 4 days of computation (including manual verification time), the final dataset after QC consisted of 2 059 052 markers genotyped on 5749 unique samples. Note that the full power of the cluster is used only for the relatedness and the ethnic modules, as only a maximum of four processes were used in parallel for the other modules.

As genetic datasets are getting larger, efficient genetic data QC and clean up procedures are required. *pyGenClean* ensures quick, customizable and traceable results with datasets of any size.

*Funding*: This work was supported by the Montreal Heart Institute Foundation, Genome Canada and Genome Quebec.

*Conflict of Interest*: none declared.
